# Non-urgent patients in emergency centres in Western Cape district health services

**DOI:** 10.4102/safp.v67i1.6116

**Published:** 2025-04-30

**Authors:** Michael Pather, Robert Mash, Daniël J. van Hoving

**Affiliations:** 1 Division of Family Medicine and Primary Care, Faculty of Medicine and Health Sciences, Stellenbosch University, Cape Town, South Africa; 2 Division of Emergency Medicine, Faculty of Medicine and Health Sciences, Stellenbosch University, Cape Town, South Africa

**Keywords:** emergency medicine, primary care, access, reason for encounter, emergency centres, district health service

## Abstract

**Background:**

Non-urgent patients are frequently found in emergency centres (ECs) and contribute to prolonged waiting times, overcrowding, high workloads and reduced quality of care. The aim of this study was to explore the perspectives of lead clinicians regarding patient attendance at ECs with non-urgent conditions in Western Cape district, South Africa.

**Methods:**

An exploratory descriptive qualitative study in which semi-structured interviews with 19 family physicians and one emergency medicine specialist from 11 district hospitals and four community health centres were conducted.

**Results:**

Key reasons for non-urgent patients to attend the EC were a lack of access to primary care, poor understanding of emergencies and health services, issues related to work and transport, referral by general practitioners, being seen quicker, preference for a doctor, dissatisfaction with primary care and worries about confidentiality. The effects were seen as reduced quality of care for urgent and non-urgent patients, overcrowding, reduced staff morale, many complaints as well as abusive behaviour, manipulation of the triage system, longer triage and waiting times.

**Conclusion:**

Action should be taken to: strengthen the primary care platform through better access, coverage and quality of care; educate communities and general practitioners; harness digital technology and telehealth; change the way emergency medical services operate and ensure adequate staffing of ECs. Alternative methods for offering primary care should be evaluated.

**Contribution:**

This article discusses the reasons for, effects of, and possible solutions to the problem of non-urgent patients attending ECs. Further studies may explore the perspectives of patients and medical officers.

## Introduction

The number of patients visiting emergency centres (ECs) is increasing in several countries even though many of them do not require treatment at ECs.^1,2^ In addition, confusion exists between those patients considered to be inappropriate for EC care and who could be managed in primary care, and those considered non-urgent, but appropriate for EC care.

The top service delivery problems in ECs include overcrowding, prolonged waiting time, extended length of stay (LOS) and patients who leave without being seen (LWBS).^3^ If these problems can be addressed then an improvement in the financial burden, patient satisfaction as well as decreasing morbidity and mortality can be expected.^4^ From the health workers perspective, inappropriate visits could represent between 20% and 40% of all EC visits.^5,6,7^

In low- and middle-income countries (LMICs) medical practitioners often lack emergency medicine training.^8^ This is especially true of sub-Saharan Africa, where high patient loads are contributing to high mortality in ECs,^9^ that is also generally higher than in high income countries.^10^

In South Africa, ECs of district hospitals are often the first point of care and in some rural settings as few as 2% are triaged as actual emergencies.^11^ Evaluation of primary care performance suggests access is still an important issue, particularly after-hours and on weekends, and for people who are working.^12^ An improved understanding of the reasons to seek care at the EC and not ambulatory primary care services is crucial.

Emergency centres are often overcrowded with significant patient burdens. The South African Triage Scale (SATS) is currently used in the Cape Metropole to guide clinical decision making and to predict mortality.^13^ In the Cape Town Metropole, patients are triaged by a trained nurse who rank them according to the SATS. Patients who are least urgent receive a triage score of two or less according to the SATS, indicating that they are non-urgent.

Timely treatment of patients with high SATS (such as very urgent [orange] and emergency [red]) is essential to reduce mortality.^14^ However, patients with low scores often present at ECs with non-urgent conditions, which could be dealt with in the hospital outpatient departments or in primary care. Heavy patient loads in ECs often detract from the overall quality of care and are associated with burn out in medical practitioners.^15^

Little is known concerning the factors associated with attendance at ECs for patients with non-urgent conditions in LMICs, including South Africa. An improved understanding of the reasons that lead patients to seek assistance at ECs is of fundamental importance.^9^ The aim of this study was to explore the perspectives of lead clinicians regarding patient attendance at ECs with non-urgent conditions in the Western Cape province, South Africa.

## Research methods and design

### Study design

This was an exploratory descriptive qualitative study using individual semi-structured interviews.

### Study setting

The Stellenbosch University Family Physician Research Network (SUFPREN) is a practice-based network of family physicians (FPs) working in the public sector.^16^ These FPs are located at 10 public sector district hospitals and 7 primary care facilities across the whole Western Cape province. In the Cape Town metropolitan health district, the network covers the East Metro with two substructures (Khayelitsha-Eastern and Northern-Tygerberg). The research question was selected and prioritised by the members of the practice-based research network.

Each district hospital and each 24-h community health centre (CHC) have an EC. The EC is staffed by interns, community service doctors and medical officers, and the FP acts as the consultant to the medical team. Community health centres serve a geographically defined population and district hospitals have a catchment area that includes several primary care facilities.

The SATS is based on identification of key discriminators to allocate the level of urgency.^17^ For example, emergency patients (classified red) might not be breathing, in cardiac arrest or hypoglycaemic. Very urgent patients (classified orange) might have altered consciousness, chest pain or acute focal neurology. Urgent patients (classified yellow) might be pregnant with vaginal bleeding, bleeding that is controlled or abdominal pain. Once these discriminators are considered then the Triage Early Warning Score (TEWS) is calculated based on the vital signs (mobility, respiratory rate, heart rate, temperature, level of consciousness, presence of trauma). The TEWS can also allocate a patient to an appropriate level of urgency: red (TEWS > 7), orange (TEWS 5–6), yellow (TEWS 3–4). Those with a TEWS of 0–2 are seen as non-urgent patients who require routine care. The guideline recommendations target times for attending to different categories of patients: red (immediate); orange (within 10 min); yellow (within 1 h); green (within 4 h).

### Study population

The study population comprised of FPs in charge of ECs at four 24-h CHCs and 10 district hospitals within the SUFPREN. The FP in charge of the EC at each facility was selected for interview. One large metropolitan district hospital was also included and an emergency medicine specialist was interviewed.

### Data collection

The researcher conducted individual semi-structured interviews with these lead clinicians in 2022. An interview guide explored the underlying issues for patients attending ECs with non-urgent problems. The opening question was ‘Why do patients attend with non-urgent issues at your EC?’. Further topics that could be explored were: the impact on quality of care and the functioning of the EC; the reactions of patients to being triaged green and having to wait; how the EC had responded to the problem and any innovations; and any suggestions for how the issue should be handled in the future. Interviews were recorded and conducted face-to-face or virtually, in English, and lasted between 30 min and 60 min.

### Data analysis

Each recorded interview was transcribed verbatim and checked for accuracy against the recording. The transcript was also sent back to the interviewee for member checking. The second author (R.M.) analysed the data according to the following framework method and with the assistance of ATLAS.ti software:^18^

- Familiarising: The researcher read the transcripts and identified key issues that could be coded.
- Coding index: The researcher defined codes based on step 1 and organised them into categories.
- Coding: The researcher coded all the data using the coding index. Additional codes were generated if necessary.
- Charting: Reports were created in ATLAS.ti based on code families that brought all the data together in one document. Codes were organised into families based on the categories from step 2.
- Interpretation: The data in each report were interpreted to identify themes and any relationship between themes.

### Trustworthiness

The interviewer (M.P.) was an academic FP, based at the university, with expertise in qualitative interviewing. He did overtime at an EC that was not part of the study, but this also gave him insights into the functioning of ECs. He knew the FPs in the network but had no hierarchical relationship to them within the Department of Health and Wellness. The analyst (R.M.) was also an academic FP at the university. He also has experience in qualitative analysis and knew the FPs in the network. He was not working at an EC and came to the data with an open mind. The themes were presented to the FPs in a workshop so that they could be validated, and the FPs could discuss the findings.

### Ethical considerations

Ethical approval to conduct this study was obtained from the Stellenbosch University, Health Research Ethics on 06 December 2021. The ethical clearance number is N21/10/109.

## Results

In all, 19 FPs and an emergency medicine specialist were interviewed from eight district hospitals in the rural health services and seven facilities in the metro health services (four CHCs and three district hospitals) (Table 1). In facilities with two FPs, they were interviewed together.

**TABLE 1:** Characteristics of family physician interviewees.

Interview ID	Health service	District or substructure	Type of facility	Number of interviewees
1	Metro	Northern-Tygerberg	Community health centre	2
2	Rural	Cape Winelands	District hospital	2
3	Metro	Khayelitsha-Eastern	District hospital	1
4	Metro	Northern-Tygerberg	Community health centre	1
5	Metro	Khayelitsha-Eastern	District hospital	1
6	Metro	Khayelitsha-Eastern	District hospital	1
7	Metro	Khayelitsha-Eastern	Community health centre	1
8	Metro	Northern-Tygerberg	Community health centre	1
9	Rural	Overberg	District hospital	2
10	Rural	Garden Route	District hospital	1
11	Rural	Garden Route	District hospital	1
12	Rural	Garden Route	District hospital	2
13	Rural	Cape Winelands	District hospital	2
14	Rural	Cape Winelands	District hospital	1
15	Rural	West Coast	District hospital	1

ID, identifier.

The main themes and sub-themes are presented and illustrative quotations are provided in Tables 2 to 4. The main themes are:

- The proportion of non-urgent (green) patients in the EC varies widely.
- Reasons why people with non-urgent problems attend the EC.
- The effects of people with non-urgent problems on the EC.
- Solutions to the problem.
- Could patients who triage green be emergencies.

**TABLE 2:** Illustrative quotations for reasons why people with non-urgent problems attend the emergency centres.

Sub-themes	Illustrative quotations
Lack of access to primary care services	‘I think that is probably the biggest driver of why patients attend EC inappropriately. So, at this stage, the waiting list to see a doctor at the clinic might be anything between three weeks to six weeks, depending on the on the complaint and the clinical condition’. (Rural, Garden Route, district hospital)
Patients’ understanding of the health system	‘I think it’s also just the fact that patients are not really educated as to what the EC really caters for, so, they will come with anything and that’s probably because of a lack of education in the whole community’. (Metro, Northern-Tygerberg, community health centre)
You might be seen faster at the EC	‘You’re guaranteed to be seen on Saturday morning by a young, energetic, fresh doctor who’s going to see you nicely now’. (Metro, Khayelitsha-Eastern, community health centre)
Work-related issues	‘What impacts is that a lot of the persons won’t get paid if they have a clinic attendance note. So, if they go to the clinic and they get a clinic attendance note from the clinic, they will be allowed to not be at work that day, but they won’t be paid for that day’. (Rural, Cape Winelands, district hospital)
Transport-related issues	‘We have a lot of farm workers. And they don’t have access to transport, not all of the farms would give them private transport. So there’s a lot more transport availability over weekends going to town. As well as they know that if they phone the ambulance after hours for a complaint that might be a way of getting to care’. (Rural, Cape Winelands, district hospital)
Referrals from GPs	‘Some GPs will send patients with a referral letter to come to the emergency unit. Bring the letters if it’s something emergency, Sister will triage put it in the yellow box. When you consult with the patient you will discover that the patient hurt his back a few weeks ago, a few days ago, and the GP referred him as an emergency’. (Metro, Northern-Tygerberg, community health centre)
Patients preferred to see a doctor	‘Another reason might be that patients often want to see a doctor rather than a nurse, and the PHC system, the way it’s set up in our two subdistricts, is that the first person that you’re going to see when you go to the PHC clinic is to see a PHC sister, a nurse’. (Rural, Garden Route, district hospital)
Patients were dissatisfied with primary care	‘Or sometimes there’s patients that have been to the clinic a number of times, and the problem has not been addressed appropriately and out of desperation are coming to the hospital where they think they are likely to be further investigated’. (Metro, Khayelitsha-Eastern, district hospital)
Patients were worried about confidentiality	‘Or it’s a confidentiality issue where the aunt is working at the clinic and they don’t want to show the aunt their drops [gonorrhoea] so they come to the hospital where there’s no one there they should know’. (Rural, Overberg, district hospital)

GP, general practitioner; EC, emergency centre; PHC, primary healthcare.

**TABLE 3:** Illustrative quotations on the effects of people with non-urgent problems on the emergency centres.

Sub-themes	Supportive quotations
Reduced quality of care	‘So if you think of the small little emergency unit, where the waiting area is almost in the emergency unit, then doctors and nurses can’t concentrate on what they’re doing, giving medications, mistakes increase, you forget to do something because somebody’s bothering you the whole time’ (Rural, Cape Winelands, district hospital).
People complain and are abusive	‘The green patients are the ones that make a lot of noise outside the EC, they are the ones that have become very abrupt, rude and abusive and fight outside of the EC which then disrupts everyone’ (Metro, Northern-Tygerberg, community health centre).
People fabricated and manipulated their symptoms	‘They will bring the patient in a wheelchair, and the patient will, be lying in the wheelchair not even sitting up or not wanting to talk, whatever. So, then they’ll triage the patient, as soon as they triaged the patient and the patient will get out of that wheelchair and go sit in the chair’ (Metro, Northern-Tygerberg, community health centre).
Long waiting times	‘Well, it can be 24 hours. I’ll have to look at our average waiting time, but I think our average for orange patients is two to four hours and they’re supposed to be seen within ten minutes. Yellows are supposed to be seen within the hour and our average time there is probably about six hours. So green patients should be seen within four hours, but it’s not unusual for a green patient to wait 16 plus hours’ (Metro, Khayelitsha-Eastern, district hospital).
Impact on staff morale	‘I think it’s that mental pressure, knowing that its full, like just seeing full boxes and so many patients waiting, like you feel you have this obligation to help everybody, but then at the same time, you know, this portion of patients I know I’m not going to get to’ (Metro, Northern-Tygerberg, community health centre).
Unable to provide effective and efficient care	‘For those patients we also don’t have all the medication, since we don’t have a pharmacy, we just have emergency stuff so often if they’ve got problems, they need to follow up anyway at the clinic or come back the next day for medicines’ (Rural, Overberg, district hospital).
Issues with space and infrastructure	‘There was no space and then we actually had an ectopic once that I had to go fetch outside. We had an appendix which was waiting on a drip outside, before we changed, we move the EC now, but it’s still not ideal’ (Rural, Cape Winelands, district hospital).

EC, emergency centre.

**TABLE 4:** Illustrative quotations on solutions to the problem and triage.

Themes and sub-themes	Supportive quotations
Communicate with the community	‘Yeah, so the things that we’ve already done is that we’ve advertised to the community how triage works. So that’s assisted us quite a lot. The people are quite well. They understand the triage system most of the time’ (Rural, Garden Route, district hospital).
Give better explanations and feedback in the EC	‘So, we have posters up, and nurses and doctors try to explain how it works and that it is not a “first come, first served” system. Green patients might have to wait quite some time and so we do explain that and explain that this hospital is for emergencies and not for all medical problems’ (Metro, Khayelitsha-Eastern, district hospital).
Defer patients from the EC	‘Yeah, the green patients get triaged, and if they’re really green, they get a letter to go to the clinic in the day with their observations and the letter and they go to the clinic and the clinic will help them so that we’re deferring’ (Rural, Overberg, district hospital).
Provide primary care in the EC	‘Yeah, I think the biggest thing would probably be the primary health care nurse and she generally works a four-hour shift on a Saturday and a four-hour shift on a Sunday. And that is from 12:00 to 16:00, which is sort of the time when EC gets busy’ (Rural, Garden Route, district hospital).
Improve access to primary care – extend opening times	‘The clinics, I mean, ideally the clinics need extended hours and even a Saturday morning clinic. I think that is the way that it should go’ (Rural, Overberg, district hospital).
Improve the quality of primary care	‘There is talk that more of these facilities will be taken over by the province and will then provide the comprehensive package of care. But we’re not there yet, so that will improve things in future’ (Metro, Khayelitsha-Eastern, district hospital).
Outreach to private GPs	‘[*XXX*] has gone on an active process in engaging with clinics, engaging with private GPs, because what’s happened is we’ve realised that a lot of the green patients for example is referred in by general practitioners because they don’t know what else to do with the patients’ (Metro, Khayelitsha-Eastern, district hospital).
Harness digital technology	‘We tried things like having the Vula App where nursing at the clinics, because all our clinics are nursing driven. If they wonder whether they should send a patient to the EC or they actually just want doctor advice they often Vula us and we can say no, the patient is not for emergency or do this and this where they might have probably sent the patient to EC and it might have been a green or a yellow. And they’re actually just needed advice’ (Rural, Cape Winelands, district hospital).
Change the approach to emergency medical services	‘But I know that clinical associates would actually be a great asset not only to the hospital, but also maybe to an EMS [emergency medical services] system, where if the EMS arrives at say for instance a house now where they are being called to, they can probably make an assessment there. And if they are, are not comfortable in making that decision, that they could have a direct line to the doctor who is on call to say, okay yeah, let’s bring that patient in or no, it doesn’t sound that serious’ (Rural, Garden Route, district hospital).
Increase staffing levels or change the staffing mix	‘Get more doctors … [*laughing*] … employ more doctors. That would be amazing if we could employ more doctors, I think just to be able to, because if you do have quite a big team, then you’re able to maybe allocate one or two doctors to see just the greens’ (Metro, Northern-Tygerberg, community health centre).
Have more facilities to serve the needs	‘Because the thing is, the community is a huge community. Like, we don’t even just cater for [*XXX*], we are catering for the whole of [*XXX*], it’s a very, the area’s quite broad. So yeah, so I feel that like, definitely another facility or two’ (Metro, Northern-Tygerberg, community health centre).
Effective leadership and teamwork	‘We are one generalist, family medicine driven team. So we allocate resources, so I’m working this weekend, when I get here tomorrow I will see where is the need, who needs to do what, where, when. I will be able to see how many green patients are waiting, how many yellows, and I think the biggest challenge people have is a rigid mind-set and the inability to adapt to what the situation needs’ (Metro, Khayelitsha-Eastern, district hospital).
Are patients that triage green emergencies	‘They can triage green, but it doesn’t mean they shouldn’t be here. I’m trying to think now, we had a young patient with abdominal cramps that triaged green. After waiting too long, they went to a GP and came back dead-on arrival on the same day’ (Metro, Khayelitsha-Eastern, district hospital).

Note: The name mentioned during the interview has been redacted for confidentiality according to ethical guidelines and indicated with [*XXX*].

GP, general practitioner; EC, emergency centre.

### The proportion of green patients in the emergency centres varies widely

Most respondents reported that 20% – 30% of their patients in the EC were triaged as green. One district hospital in an urban area reported that only 10% (8 patients per night shift) were triaged green, while a semi-rural district hospital reported it could be as high as 40%.

### Reasons why people with non-urgent problems attend the emergency centres

#### The lack of access to primary care services

One of the main reasons for attendance at the EC was the lack of access to primary care services. Many primary care facilities were not open on weekends or evenings, and only operated during office hours (08:00–16:00). Outside of these times, the EC was the only option. In rural areas, the clinics might not be open every weekday. In several areas there was no 24-h primary care facility, and an EC at the district hospital was the only option.

Most primary care facilities had started an appointment system for patients, but these were oversubscribed; therefore, appointments might only be available after 4–8 weeks. In some cases, there were only appointments for the people with chronic conditions and not acute problems. There was a perception that some communities did not trust appointment systems and did not cooperate with them. In addition, patients that were seen in primary care with a new problem may deteriorate, or may be worried about new symptoms and find it difficult to access primary care again.

Many primary care facilities had a walk-in service for unbooked patients. Patients were triaged briefly and the number of unbooked patients allowed was then aligned with the number of staff and capacity on that day. The remaining patients were asked to try again the next day or make an appointment. In one or two cases, the clinics did not have a system for unbooked patients to be seen. In one clinic, the unbooked patients were consulted briefly and had to enter the appointment system if they needed investigations or a longer consultation. Many patients were therefore deferred or not willing to wait for an appointment and then chose to present at the EC

#### Patients’ understanding of the health system

People might not differentiate between primary care and emergency services and the types of problems that belong in these different settings. Likewise, they may not differentiate between primary care and district hospital services, and how these levels of care differ. People’s interpretation of symptoms and what constitutes an emergency could also differ from the health professionals’ perspectives. The understanding of rules and regulations for different problems and levels of care in the minds of healthcare workers was not shared by community members. In addition, once someone has been assisted at the EC for a non-urgent condition, this may reinforce the same help-seeking behaviour in the future.

#### You might be seen faster at the emergency centre

In certain facilities and at certain times, a patient that triaged green might be seen faster than at the primary care services. For example, early on a Saturday morning or late in the evening the EC might be quiet, and you would be seen quickly by a doctor. In addition, you might not have to take a day off work.

#### Work-related issues

Many people had jobs that did not provide benefits and operated on a no work, no pay basis. If they attended primary care during the day and provided a sick certificate, they would still not be paid. Workers, therefore, would prefer to be seen in the evening or weekends and the only option available might be the EC. Likewise with children, if the parent was working, they may wait until after work to come to the EC or may only discover the child is sick when they return home from work. Similarly, parents may be reluctant to take children out of school and prefer to attend in the evening or weekend. By contrast, some patients attend the EC just to obtain a sick certificate for the next few days.

#### Transport-related issues

Many people did not have their own transport. For example, farmworkers might only have access to transport to the town on weekends. At this point primary care was closed, but the EC was open. Others may only have transport when someone comes home from work in the evening.

In addition, some facilities may not have easy access to public transport, such as taxis. Patients will then phone the ambulance and be taken to the nearest EC and not primary care, even if it was open. One respondent reported that ambulances would not take someone to a primary care service after 14:00. Once at the EC, patients might struggle to get transport home, especially at night, and therefore would have to stay in the EC. This could potentially add to the problem of overcrowding.

#### Referrals from general practitioners

Private GPs often referred patients to the EC with non-urgent problems. This might be because they did not know how to refer patients to other public sector services, and this was the easiest gateway into the system.

#### Patients preferred to see a doctor

Most consultations in primary care were with a nurse and many patients would prefer to see a doctor. At the EC, patients are much more likely to see a doctor and to have access to additional investigations such as X-rays or blood tests. Care from a doctor was perceived to be better than that of a nurse practitioner. At primary care facilities you might need to schedule an appointment to see a doctor at a later date. Patients might also perceive that clinic doctors were inferior to hospital doctors, even though in rural areas they were the same people.

#### Patients were dissatisfied with primary care

There were also patients that had attended primary care multiple times with the same problem and felt dissatisfied with the care. Maybe their condition was not cured, diagnosed or they had a poor experience. Rather than asking for referral they came to the EC to get a second opinion or thought the quality of care would be better. Patients sometimes came from other provinces for the same reason.

#### Patients were worried about confidentiality

For a small group of patients, maybe in smaller rural communities, they were worried about confidentiality at the local clinic. The nurse might also live within the community, and there were concerns they would disclose their diagnosis to others.

### The effects of people with non-urgent problems on the emergency centre

#### Reduced quality of care

The pressure of large numbers of people with non-urgent problems made the doctors work faster and possibly cut corners with those who were acutely ill. For example, you might take a shorter history or not check on previous test results because of the lack of time. Patients with non-urgent problems interrupted consultations to complain that they had been waiting too long, and therefore disrupted clinical reasoning and treatment. This could lead to errors and omissions. It was difficult to abruptly leave a patient who was not an emergency to attend to a more urgent problem.

#### People complain and are abusive

There was complete agreement that the bulk of complaints in the EC came from the people who were triaged green. They had to wait for a longer period, saw people that came after them being attended to first and were well enough to complain. Many people did not simply complain but became verbally and physically aggressive with the health professionals. In a few cases, this was fuelled by alcohol or other substances. Some lodged formal complaints with the facility or even the department of health. At times, they also confronted other patients who were called first.

Several doctors reported that they tried to avoid seeing these abusive patients and might also become rude or unfriendly. Patients were often able to exit the waiting room and confront doctors in the EC itself. Patients might also misinterpret what they saw, as doctors accessed the results of investigations on their cell phones or were speaking to colleagues at the referral hospital. Patients might think they were checking their social media or chatting to friends. Dealing with upset and angry patients could take time away from other patients that needed more urgent attention, and ironically prolong the waiting time for all patients. Patients’ frustrations were amplified if they had waited a long time before being told that they were not going to be seen.

#### People fabricated and manipulated their symptoms

Some patients had worked out how to manipulate the triage system to be seen quicker. By complaining of collapse, chest pain, palpitations, or shortness of breath, for example, they were triaged yellow or orange instead of green. During the consultation, the doctor would realise that their complaint and triage score did not align. However, it was usually easier to see and treat the patient than potentially argue with them over the appropriate level of urgency.

#### Long waiting times

Having many patients with non-urgent problems could prolong the time taken to triage all patients and might delay the identification of those with urgent problems. Patients that triaged green often had to wait many hours to be seen, and much longer than the recommended norms in the guidelines. The psychological burden of knowing that these patients were waiting outside might be difficult, and doctors could try and squeeze them in between more urgent patients.

#### Impact on staff morale

Trying to cope with high numbers of patients who did not have emergencies, and were often complaining could erode staff morale. This could contribute to stress, being overworked, burnout, and a lack of empathy and patience. This psychological load might be compounded by unsupportive managers who wanted staff to work quicker. When doctors had to deal with a high stress situation such as a resuscitation or death of a patient, it was difficult to immediately turn round and start seeing non-urgent patients.

#### Unable to provide effective and efficient care

Although patients attended the EC for a wide variety of primary care problems, the EC only had an emergency pharmacy supply and not the full range of medications. This meant that even if patients saw the doctor, they would still have to attend the primary care services to get their treatment.

In addition, primary care patients often had multiple complex problems, while the EC is orientated towards dealing with just the one emergency. Patients might not get the comprehensive assessment that would be expected in primary care. The temptation was to treat their symptoms and defer to the primary care services. The EC was also not orientated towards routine screening or preventative activities such as tuberculosis (TB) or cervical cancer screening. Providing primary care in the EC from a doctor was also more expensive, and patients might be over investigated in the hospital environment when compared to primary care.

#### Issues with space and infrastructure

Some rural facilities had very limited space in the EC and waiting room and became congested when there were too many people with non-urgent problems. Sick patients could even end up in the car park or outside. Patients could also congest the EC while they waited for a bed in the hospital because the wards were full.

### Solutions to the problem

#### Communicate with the community

Respondents suggested media campaigns (e.g., radio, print, social media, television) in the local community and languages to explain the triage process and what happens in an EC. In particular, the waiting times for people with non-urgent problems could be discussed. could be discussed. People’s expectations and help-seeking behaviours need to be adjusted. People should be informed about what is an emergency and how to make better use of services during the day. People should understand the different levels of the health system and how they can use the limited resources better. They can also be informed on how to make use of over-the-counter medication or home remedies, rather than coming for paracetamol or oral rehydration solution. Such community engagement needs to be consistent over time to have an incremental effect. One should target key community members to give health advice such as elders or community health workers. However, the facilities struggle with effective community engagement and media initiatives are often centralised.

#### Give better explanations and feedback in the emergency centre

Many ECs have put up posters to explain the triage system, but this also assumes patients can and will read them. One facility had made a video that played on a loop. Some facilities had a queue marshal who could also explain what was going on and give feedback on likely waiting times. Having a dynamic display should now be possible based on the digital Health Emergency Centre Trauma Information System (HECTIS). Waiting times and the number of people in the queue in the different triage categories can be made visible. Patients were not always aware of the triage system and assumed that they would be seen on a first come, first served basis. Respondents reported that the same triage system was not implemented across all types of health facilities.

#### Defer patients from the emergency centre

A general principle was that every patient should be triaged. During office hours in primary care facilities, patients who presented to the EC and triaged green could be taken across to the primary care services. Even at district hospitals a doctor might defer patients back to primary care during office hours. At some facilities after hours patients would be given the option of a letter to their primary care service or an appointment to the outpatients department. It was a good practice to explain promptly that the waiting time would be long and that there were other options available to patients. Waiting hours to see the doctor and then being deferred, resulted in complaints and frustration. No one was forced to leave however, and the one doctor who attempted to do this was met by opposition from the nursing staff. Respondents reported that deferring a patient should be done by a doctor and there was not always a spare doctor to do this. Patients could be given symptomatic treatment until they were assessed more thoroughly at the clinic. Some doctors explained that it took longer to negotiate a deferral of a patient than to consult them. Accommodating non-urgent patients in the EC could however, reinforce this help-seeking behaviour in the future.

#### Provide primary care in the emergency centre

Many facilities were employing nurse practitioners in the EC for extended hours particularly on weekends. This was to see the primary care patients. These arrangements were somewhat ad hoc and relied on the willingness of nurses and availability of funds. This also gave the message that even if you come to the EC with a minor problem, you will see a nurse and not a doctor. During office hours, one facility reported that they had a gateway primary care clinic on the same site. In one facility, the nurse practitioner felt it was not the right setting for her, and she was replaced by a ‘see-and-treat’ doctor.

#### Improve access to primary care – Extend opening times

Primary care facilities should manage walk-in patients. Primary care services need to look at ways of extending their services into the early evening and weekends. Patients should be able to get help in the afternoon as well as the morning. It would be more aligned with the needs of people and the health system to provide additional staff in primary care, than in the EC to manage primary care problems.

#### Improve the quality of primary care

The technical quality of care and patient experience should be improved to build confidence and trust in primary care. This may mean further inclusion of doctors and FPs in the primary care team. Having more access to a doctor in primary care may also reduce attendance at the EC. Some local government clinics did not offer a comprehensive package of care and this needs to be addressed. Primary care should ensure a mixture of male and female staff to cater for gender preferences (e.g., Xhosa men may not be willing to see a female nurse). This would help prevent people bypassing the primary care system.

#### Outreach to private general practitioners

Active engagement with private GPs can help them access the health system more appropriately via outpatients or primary care services.

#### Harness digital technology

Nurses in primary care could use technology such as the Vula App to liaise with doctors in the EC and get advice on whether the person is an emergency. This can also be extended to GPs to help them better access the public healthcare system and outpatient appointments. Digital technology such as WhatsApp can also be used to streamline the process of obtaining an appointment in primary care. One facility reported that the EC had gone digital, and the clinical notes could be accessed from primary care, thus improving the coordination of care. Some people mentioned the use of telehealth and teleconsultations in other countries as a possible solution to help people self-manage minor problems.

#### Change the approach to emergency medical services

Ambulances should not be afraid to take patients with non-urgent problems to a primary care facility. Emergency medical personnel could be upskilled to better triage patients (e.g., employ clinical associates) and liaise with the doctor on call before bringing a patient.

#### Increase staffing levels or change the staffing mix

Having more doctors in the EC would allow some of them to be allocated to seeing the patients triaged as green. Usually, because of resource constraints, it is not possible to allocate doctors to focus on particular triage streams. There was an impression that district hospitals had larger staffing levels in the EC. The increased availability of interns in family medicine and primary care was welcomed. One facility made use of medical students to assist with the patients triaged green.

Employing more mid-level professionals in the EC to assist with reading radiographs or performing procedures might be cost-effective. A few respondents suggested that employing clinical associates could assist the doctors as they can work clinically to perform procedures (e.g., suturing). Nurses may also assist in this way in some settings.

#### Have more facilities to serve the needs

In some facilities, the population in the drainage area was perceived to be too large and increasing over time. Additional facilities were needed to provide both adequate primary and emergency care. One suggestion was to expand access to primary care by increasing the number of small clinics and expanding community-based services. In one rural area, the expansion of private sector facilities may relieve the pressure after hours on the public sector EC.

#### Effective leadership and teamwork

Family physicians appeared to have a good knowledge of primary care and the broader health system. They used this knowledge to tackle the problem with more systems level thinking, while other specialists might just focus on what was happening in the EC. Sometimes people adopted rigid roles in the clinical team and were not willing to adapt to the situation, for example, being willing to see people who are triaged green if that was the need at the time. A few facilities had a rule that all patients arriving during your shift needed to be managed by that team and not handed over. This was perceived to incentivise doctors to work more efficiently, or they would have had to stay longer.

In primary care, doctors often see the patients with chronic diseases and expect the nurse to see the new undifferentiated problems. Sometimes the undifferentiated patient requires a higher level of clinical reasoning and a doctor should be available to assist. This could reduce referrals to the EC.

### Are patients who triage green emergencies?

There were many comments on the accuracy of triage and the implications. The overall sense was that it worked well for most patients but there were notable exceptions. Mistakes could happen in both directions. Patients who should be triaged green could be found in the yellow box. One facility believed that 60% of the yellow patients were green. On the other hand, some of the green patients did have problems that were appropriate to the EC or should have been triaged with more urgency. There were many stories of patients who were triaged green who had died in the waiting area or after being sent home. Overall, it was better to over assess than under assess the urgency. Suggesting to a patient that they be deferred to the next day or primary care, needed to be performed carefully with appropriate safety netting and by a more experienced clinician.

Errors in triage might be because of over reliance on the vital signs and insufficient clinical experience among junior nurses to assess the more subjective discriminators. Errors might also occur from rushed and careless triage with a desire to process the queue and go home. Although triage is performed by nurses, it can be helpful to have support from a doctor who can spot patients who need to be upgraded from green to yellow or decide on deferring the patient.

The 24-h ECs in CHCs may be more tolerant of people triaged green as they are primary care facilities. Sometimes people who were triaged green were scored as yellow or even orange so that they could be seen quickly. This might happen because the nurse knew the patient or they were a family member. The more subjective discriminators may be manipulated by the patient or used as levers by the nurse, for example, if the person is getting upset or has been waiting very long.

Although the guidelines recommend that people be re-triaged after 4 h, this almost never happens. This meant that people were not re-assessed to see if their condition has changed. Almost all the ECs did not have capacity to do this. Even when quality improvement projects addressed this issue, they were not successful. Sometimes doctors would go through the pile of folders to try and spot patients who needed to be re-assessed. Nurses might notice someone deteriorating in the waiting room on the video monitor or patients might raise the alarm. One facility reported that re-triaging a patient could also reduce complaints as well as improve patient safety.

The HECTIS allowed the doctors to monitor the patients who were waiting and review the triage. This also allowed doctors to spot patients who were wrongly triaged or had waited too long. Doctors could also ask for tests to be performed while the patient was waiting. In addition, the system analysed the vital signs and discriminators and improved the allocation of the correct triage colour. Doctors could monitor the situation in the EC from anywhere in the hospital. Managers can also create accurate statistics on waiting times and workload. The system also prompted doctors when patients had waited too long or needed to be re-triaged.

Adherence to the guidelines for waiting times varied substantially between facilities. One facility reported that green patients might wait 16–24 h to be seen and many absconded or went home to sleep. Other facilities reported that they were mostly seen within 4 h. Often the EC had so many patients categorised as red, orange, and yellow that the staff never got to the green ones. One patient explained that it would take them 4 days to earn enough money to see someone in private practice and therefore waiting 4-h was worth it.

The time waiting to be triaged was a key factor, as the triage nurse could give the patient information on how long they might have to wait. It was important to give an honest assessment immediately and suggest other options rather than making them wait and be told later that they were unlikely to be seen. If patients had waited long to be triaged and be told that they were not supposed to be there, then this increased their irritation and anger.

Most facilities had regular training of the nursing staff on how to triage and gave feedback on any errors. Training needs to be ongoing as new staff regularly joined the team or others were rotated into the EC. Sometimes an error in triage and a patient safety incident would also prompt training.

## Discussion

### Summary of key findings

**FIGURE 1:**
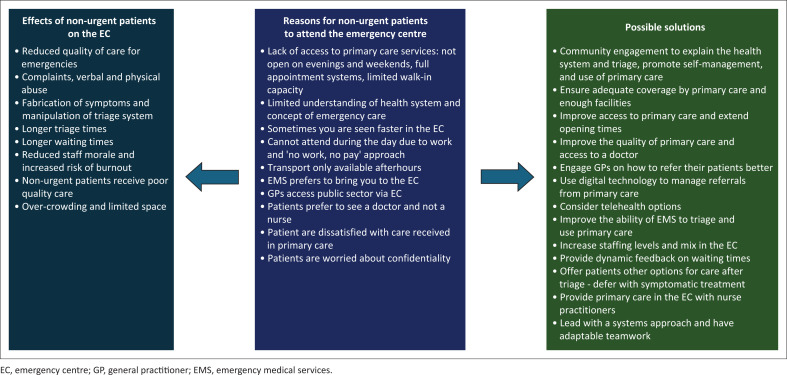
Summary of key findings.

Figure 1 summarises the key findings in terms of the reasons why people attend the EC with non-urgent problems, its effects on the EC and possible solutions. While patients who were triaged ‘green’ were labelled as non-urgent, there were clearly situations where this was not the case. The SATS was designed to rank the urgency of the patient and not to differentiate between emergencies and non-emergencies or to determine who could be deferred and not seen at all.

### Discussion of key findings

Many of the reasons for attending the EC are related to issues with the primary care platform such as access, quality of care, trust in the nurse practitioner and behaviour of private sector general practitioners. The hypothesis of many FPs was that strengthening the primary care platform would reduce utilisation of the EC by non-urgent patients.

Access is consistently identified as a weakness of South African primary care and may be related to opening times, appointment systems and capacity.^12^ In high income countries, improving after-hours access to primary care did reduce use of the EC, but the effects were mixed and evidence limited. The effects may be context dependent and thus local evidence is required to show the benefit.^19^ However, another review of GP co-operatives (first contact nurse practitioners with general practitioner (GP) supervision operating after-hours) found good evidence for a reduction in EC utilisation.^20^

Greater involvement of doctors and FPs in the primary care team has been recommended as a way of improving the comprehensiveness and quality of care.^21,22^ More public–private partnerships and coordination of private and public sector care could improve GP behaviour. The introduction of national health insurance could also expand the available primary care platform to all South Africans and reduce pressure on the ECs. Currently, budget cuts and austerity measures are shrinking capacity and resources, and make it difficult to strengthen primary care.

Providing more primary care in the EC was also suggested as a solution. This could be a simpler and cheaper solution than changing after-hour access to primary care facilities. For example, employing nurse practitioners or medical officers in the EC could be cheaper than paying all the staff needed to extend the opening times of primary care facilities. However, evidence from high income countries is inconclusive on the effectiveness or safety of this approach.^23^ There is also a risk that installing such services will result in provider-induced increased demand that inadvertently changes health-seeking behaviour.^24^ This could actually worsen the problem of overcrowding and non-urgent attendance at the EC.

The provincial commitment to community-orientated primary care includes a need to improve community engagement and the possibility of community health workers disseminating information to local communities. This could be leveraged to enhance people’s understanding of the EC, the triage system and their options for healthcare.

Several digital solutions were mentioned that could assist with reducing the number of non-urgent patients in the EC. The Vula App has been shown to help with referrals from primary care to district hospitals.^25^ The App can avert unnecessary referrals by allowing the EC doctors to give advice or offer outpatient appointments. The Department of Health and Wellness are planning to develop their own similar App. The HECTIS system also allows the EC to manage patient flow and workload better and provide more up-to-date feedback to patients on waiting times.^26^

Emergency medical services generally transport patients to the EC. In high income countries, the ambulance crew have worked with triage protocols to identify patients that do not need emergency care versus those that clearly do. The group of intermediate patients can be offered alternate care, for example, by primary care providers or walk-in clinics. The evidence is supportive of this but lacks methodological rigour, and further research is needed that evaluates patient outcomes in this regard.^27^ Family physicians suggested experimenting with such approaches in the South African context.

### Strengths and limitations

Data were obtained from FPs across all districts and from both primary care and district hospitals. Data saturation was reached with these 15 interviews. The results should be transferable within the Western Cape district and probably to similar contexts within South Africa.

### Implications

The health services should consider multifaceted approaches to strengthening primary care services such as: after-hours services, increased capacity to reduce the wait for appointments and need to defer walk-in patients; greater access to a doctor and engagement of FPs in primary care teams; education of local private GPs; additional facilities in underserved areas; and ensuring a comprehensive package of care.

The community-orientated primary care approach and local media should educate communities on self-care, how to best access the health system and how the EC operates, particularly the triage system.

Digital technology should be harnessed to improve the referral of patients to the EC and the management of patient flow within the EC as well as dynamic feedback to patients in the waiting area. For example, the Vula App and HECTIS.

Consideration should be given to EMS offering alternate pathways to care and not automatically taking all patients to the EC. Telehealth could also be an option worth exploring.

This study was based on the experience and perspectives of FPs who were in-charge of the ECs. The findings should be triangulated with the views of patients and medical officers working in the ECs. This could provide an even greater in-depth understanding of the issues.

Most of the evidence for these solutions comes from high income settings, a more methodologically rigorous and contextualised evidence of what works is also needed.

## Conclusion

Family physicians identified multiple reasons why patients with non-urgent conditions attend ECs in district hospitals and 24 h CHCs. Many of these are related to problems of access and quality in the primary care platform. Family physicians documented the negative effects that this burden places on the EC in terms of longer waiting times, reduced quality of care and erosion of staff morale. They also suggested several solutions to help resolve the issue. These included actions to strengthen the primary care platform, educate communities, harness digital technology and to change the way that EMS operate.
